# Nocardiosis in Free-Ranging Cetaceans from the Central-Eastern Atlantic Ocean and Contiguous Mediterranean Sea

**DOI:** 10.3390/ani12040434

**Published:** 2022-02-11

**Authors:** Pablo Díaz-Santana, Antonio Fernández, Josue Díaz-Delgado, Ana Isabel Vela, Lucas Domínguez, Cristian Suárez-Santana, Raquel Puig-Lozano, Carolina Fernández-Maldonado, Eva Sierra, Manuel Arbelo

**Affiliations:** 1 Veterinary Histology and Pathology, Institute of Animal Health and Food Hygiene (IUSA), University of Las Palmas of Gran Canaria, 35413 Las Palmas de Gran Canaria, Spain; pablo.diasantana@gmail.com (P.D.-S.); antonio.fernandez@ulpgc.es (A.F.); josue.diazdelgado@tvmdl.tamu.edu (J.D.-D.); cristian.suarez@ulpgc.es (C.S.-S.); raquelpuiglozano@gmail.com (R.P.-L.); manuel.arbelo@ulpgc.es (M.A.); 2 TVMDL, Veterinary Medical Diagnostic Laboratory, Texas A&M University, Amarillo, TX 79016, USA; 3 Centro de Vigilancia Sanitaria Veterinaria (VISAVET), Complutense University, 28040 Madrid, Spain; avela@ucm.es (A.I.V.); lucasdo@visavet.ucm.es (L.D.); 4 Department of Animal Health, Faculty of Veterinary, Complutense University of Madrid, 28040 Madrid, Spain; 5 Seashore Environment and Fauna, Calle Sevilla, 4, 11380 Cádiz, Spain; carogue38@hotmail.com

**Keywords:** cetacean, nocardiosis, striped dolphin, bottlenose dolphin, Canary Islands, Andalusia, Spain

## Abstract

**Simple Summary:**

Characterization, description, and geographical location of harmful bacterial agents in cetaceans are important for population surveillance and health monitoring around the world. This research compiles the pathologic features of nocardiosis in five free-ranging delphinids from the Canary Islands and Andalusia. All examined animals showed a disseminated pattern of infection with characteristic suppurative to pyogranulomatous lesions with thromboembolism in two or more organs. The obtained results provide the first record of *N. otitidiscaviarum* in cetaceans, the first account of *N. farcinica* in free-ranging dolphins, and confirmation of nocardiosis in central eastern Atlantic Ocean.

**Abstract:**

We report the pathologic features of nocardiosis in five free-ranging delphinids from the Canary Islands and Andalusia, namely four striped dolphins (*Stenella coerulealba*) and one bottlenose dolphin (*Tursiops truncatus*). All animals had a multiorgan (disseminated) pattern of infection involving suppurative to pyogranulomatous and thromboembolic lesions in two or more organs. Most affected organs were (by decreasing order) lung, pulmonary lymph nodes, liver, kidney, adrenal glands, and central nervous system. Typical intralesional and intravascular branched and filamentous bacteria were highlighted by Grocott’s methenamine silver and Gram stains. Bacterial analysis including 16S rRNA gene sequencing identified *Nocardia farcinica* in two striped dolphins and *Nocardia otitidiscaviarum* in one striped dolphin and the bottlenose dolphin. All dolphins tested (*n* = 4) for cetacean morbillivirus were negative; one dolphin had concurrent cutaneous herpesvirosis. These results provide the first record of *N. otitidiscaviarum* in cetaceans, the first account of *N. farcinica* in free-ranging dolphins, and confirmation of nocardiosis in central eastern Atlantic Ocean. These results expand the known geographic range of nocardiosis in cetaceans.

## 1. Introduction

Nocardiosis is a relatively uncommon but widely distributed and well characterized disease of humans and animals. It is caused by a ubiquitous, saprophytic, Gram-positive, acid-fast variable, filamentous, aerobic, nonmotile, non-spore forming bacteria belonging to the genus *Nocardia* (Phylum Actinobacteria; order Mycobacteriales; family *Nocardiaceae*) [1,2,3,4,5]. More than 50 of 119 documented *Nocardia* species are clinically relevant [6,7]. While *Nocardia* sp. may act as primary pathogen, opportunistic infections are more common and can develop through diverse infective routes: inhalation, ingestion, or wound contamination due to penetrating injuries with subsequent risk for potential vascular invasion and dissemination [4]. Five main presentations have been described in mammals, namely: (i) pulmonary; (ii) cutaneous, further subdivided into superficial, lymphocutaneous (subcutaneous), and actinomycetoma; (iii) nervous (without lung involvement); (iv) systemic (encompassing two or more organs); and mastitis (primarily in dairy cattle). However, presentation overlap may occur, and dissemination is a common sequela after bacterial circumvention of host’s immunological mechanisms, particularly to the central nervous system (CNS) and skeletal soft tissue [8,9,10].

In aquatic organisms, *Nocardia* is a concern in free-ranging and captive teleost and shellfish, as well as in marine mammals [1,11,12,13]. In cetaceans, nocardiosis occurs more often as a systemic form, involving two or more organs [12,13,14]. Frequently concomitant with the systemic pattern, pulmonary nocardiosis is also common and infection has been described in striped dolphins (*Stenella coeruleoalba*), short-finned pilot whales (*Globicephala macrorhynchus*), Pacific bottlenose dolphins (*Tursiops gilli*), Atlantic bottlenose dolphins (*Tursiops truncatus*), killer whales (*Orcinus orca*), and beluga whale (*Delphinapterus leucas*) [12,15,16]. Secondary nocardial mastitis was reported in an adult beluga whale [17]. To date, nocardial species known to affect cetaceans include *Nocardia asteroides*, *N. farcinica*, *N. brasilensis*, *N. cyriacigeorgica* and *N. levis* [12,15]. Here we report the pathologic features of nocardiosis in five free-ranging delphinids from Spain, specifically from the Canary Islands and Andalusia.

## 2. Materials and Methods

This study focused on four striped dolphins (*Stenella coeruleoalba*) and one bottlenose dolphin (*Tursiops truncatus*) (cases 1 through 5) stranded along the coast of the Canary Islands and Andalusia wherein pathologic examination results were consistent with nocardiosis. In four of five dolphins, microbiologic and molecular analyses confirmed nocardial infection.

Life history was based on total body length and gonadal development, including fetus/neonate/calf, juvenile/subadult, and adult [18,19,20]. The body condition was subjectively classified into good, moderate, poor, and emaciated according to anatomic parameters such as the osseous prominence of the spinous and transverse vertebral processes and ribs, the mass of the epaxial musculature, and the amount of fat deposits, taking into account the species and the life history category of the animal [18,19,20]. Carcasses were classified as very fresh, fresh, moderate autolysis, advanced autolysis or very advanced autolysis [18,19,20].

Necropsies in all cases followed standardized protocols [18,19,20,21]. Representative samples of skin, *longissimus dorsi* and *rectus abdominis* muscles, peritoneum, diaphragm, central nervous system, eye, pterygoid sac, tympanoperiotic complexes, tongue, oral mucosa, pharyngeal and laryngeal tonsils, esophagus, stomach, small and large intestine, liver, pancreas, trachea, lung, heart, aorta, kidney, ureter, urinary bladder, urethra, lymph nodes, spleen, testicle, penis, prepuce, ovary, uterus, vagina and vulva, were collected and fixed in 10% neutral buffered formalin. All these tissues were processed routinely, embedded in paraffin-wax and 5 μm-thick sections were stained with hematoxylin and eosin (H & E) for microscopic analysis. Special histochemical techniques (4–10 μm-thick sections) to better characterize microscopic changes on selected tissue sections included Gram/Twort, Grocott-Gomori’s Methenamine Silver, and Ziehl-Neelsen.

In all animals but in case 3, due to unavailability of fresh sampled tissues, sterile swabs from tonsils, tracheal mucosa, pleura and peritoneum, as well as fresh samples from lung, liver, kidney, lymph nodes, blood, and brain were collected routinely during necropsy, frozen (−80 °C) and selectively submitted for bacteriologic analysis. Samples were surface plated on Columbia blood agar plates (bioMérieux, Madrid, Spain), and incubated for 48 h at 35 °C under both aerobic and anaerobic conditions. One colony of each sample yielding growth was selected as representative isolate and subcultured for further identification and genetic typing analysis. The bacteria were identified by matrix-assisted laser desorption ionization-time of flight mass spectrometry (MALDI-TOF MS) using the BioTyper system (software version 3.1; Bruker-Daltonics, Billerica, MA, USA) as described by Pérez-Sancho et al. [22]. Identification was further confirmed by sequencing the 16S rRNA gene of each isolate as described previously [23]. The determined sequences, consisted of the almost complete 16S rRNA gene (>1400 nucleotides), were compared with the sequences of other Gram-positive species available in the GenBank database, by using EzTaxon, server ([24]; http:/eztaxon-e.ezbiocloud.net/ (accessed on 30 November 2021).

PCR analysis for detection of cetacean morbillivirus (CeMV) and herpesvirus (HV) followed published protocols [25,26]. The required permission for the management of stranded cetaceans in the Canarian archipielago and Andalusias’s coast was issued by the Secretary of State for Environment of the Spain’s Government. No experiments were performed on live animals because our work was based on dead stranded cetaceans.

## 3. Results

The total of necropsied cetaceans in both geographical locations ascent to 865 animals between 1999 and 2020 (Andalusia: 147 animals [2011–2015] and Canary Islands: 718 animals [1999–2020]) from which five animals (0.58%) were diagnosed with *Nocardia spp*. infection and included in this research. The dolphins studied were three subadult striped dolphins (two females and one male) from Andalusia (provinces of Almería, Cádiz, and Málaga, respectively), and one adult male striped dolphin and one calf male bottlenose dolphin from Canary Islands (islands of Fuerteventura and Gran Canaria, respectively) that stranded between 2001 and 2020. Biologic and stranding epidemiology data for each animal are recorded in Table 1.

**Figure 1:**
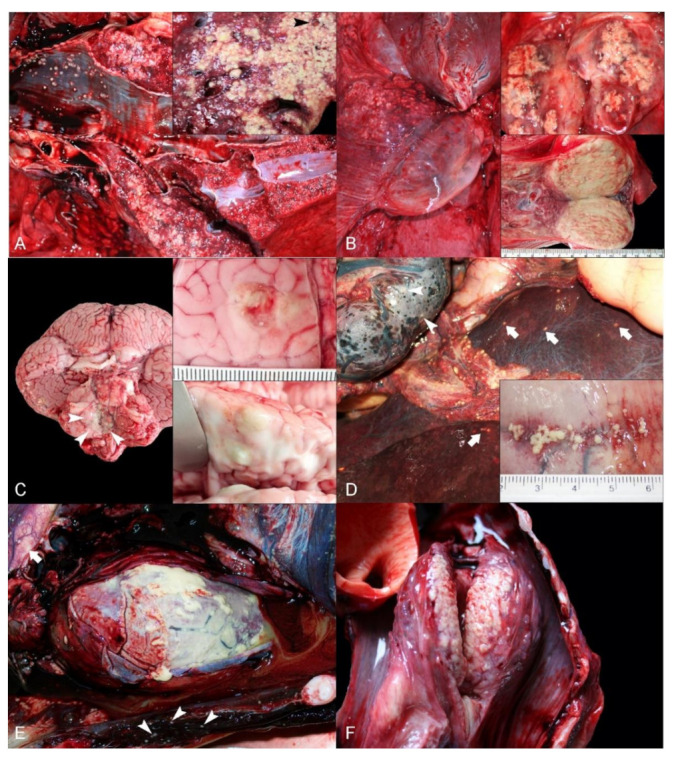
Gross pathologic findings in dolphins with Nocardia infection. (**A**) Lung; Striped dolphin. Multifocal to coalescing, pyogranulomatous bronchopneumonia with mucosal intrabronchial excrescences. Inset: Detail of pyogranulomatous inflammation obliterating lung parenchyma, bronchi, bronchioles and vessels (arrowhead). (**B**) Pulmonary lymph nodes; Striped dolphin. Right pulmonary lymphadenomegaly and pleural lymphangiectasia. The adjacent pulmonary parenchyma has pyogranulomatous bronchopneumonia. Upper inset: Detail of multifocal to coalescing pyogranulomatous and necrotizing lymphadenitis on cut surface. Lower inset: Diffuse lymph node effacement by pyogranulomatous inflammation with necrosis. (**C**) Brain; Atlantic bottlenose dolphin. Multifocal purulent exudate on the ventral surface of the metencephalon, cerebellum and myelencephalon (arrowheads). Upper inset: Cerebral cortical abscess. Lower inset: Detail of cerebral cortical abscesses expanding the white and grey matter on cut surface. (**D**) Omentum, pancreas, liver, spleen; Striped dolphin. Miliary pyogranulomas throughout the peritoneal serosae. Note pyogranulomas within the spleen (arrowheads) and liver (arrows). (**E**) Heart and pericardium; Striped dolphin. Marked, diffuse pyogranulomatous pericarditis. Note pyogranulomas within skeletal thoracic and diaphragmatic muscles (arrowheads) and periaortic serosa (arrows). (**F**) Laryngeal tonsil; Striped dolphin. Marked, diffuse pyogranulomatous and necrotizing laryngeal tonsilitis on cut surface.

Gross pathologic findings associated with nocardiosis were (by decreasing order): pulmonary pyogranulomatous lymphadenomegaly and lymphadenitis (5/5; 100%); pleural, pulmonary, and bronchial pyogranulomas (5/5; 100) (Figure 1A); cerebral abscesses (3/5; 60%) (Figure 1B); pyogranulomatous hepatitis (3/5; 60%) (Figure 1C); pyogranulomatous adrenalitis (3/5; 60%); peritoneal (serosal) pyogranulomas (3/5; 60%) (Figure 1D); suppurative pericarditis (2/5; 40%) (Figure 1E); tracheal mucosal pyogranulomas (2/5; 40%); exudative pharyngeal tonsilitis (2/5; 40%) (Figure 1F); splenic pyogranulomas (2/4; 40%); mesenteric and tracheobronchial pyogranulomatous lymphadenomegaly and lymphadenitis (2/4; 40%); renal pyogranulomas (1/5; 25 %); pancreatic pyogranulomas (1/5; 25 %), and hypophyseal pyogranulomas (1/5; 25 %) (Table 2).

Additional gross findings in these dolphins were: cutaneous traumatic intra-/interespecific (tooth rake) marks (2/5; 40%); cutaneous epibiosis by *Syncyamus* sp. and *Xenobalanus* sp. (1/5; 20%); pancreatic trematodiasis by Brachycladiidae (2/5; 40%); gastric trematodiasis by *Pholeter gasterophilus* (1/5; 20%); hepatic trematodiasis by Brachycladiidae (1/5; 20%); subcutaneous nematodiasis by *Crassicauda* sp.; peritoneal cestodiasis by *Monorygma grimaldii* (1/5; 20%); and subcutaneous cestodiasis by *Phyllobothrium delphini*.

**Figure 2:**
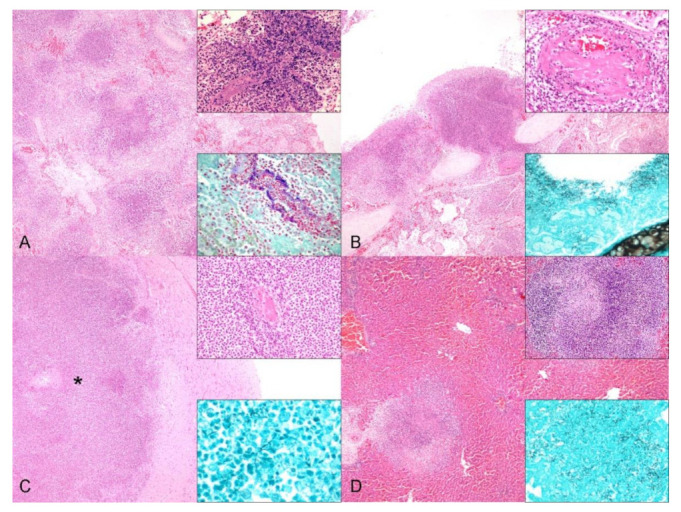
Histopathologic findings in dolphins with Nocardia infection. (**A**) Lung; Striped dolphin. The alveoli, bronchioles and bronchi are distorted and replaced by necrosuppurative exudate (low magnification). H & E. Upper inset: Detail of necrosuppurative inflammatory infiltrate targeting alveolar septa and vessels with intralesional bacteria. H & E. Lower inset: Intralesional Gram-positive filamentous branching bacteria. Gram stain. (**B**) Primary bronchus; Striped dolphin. The bronchus is coated and infiltrated by necrosuppurative exudate forming nodules that bulge into the lumen. H & E. Upper inset: Detail of vasculitis and fibrinoid necrosis within affected lung parenchyma. Lower inset: Filamentous bacteria in the inflamed bronchus are highlighted by Grocott-Methenamine silver (GMS). GMS (**C**) Cerebral cortex; Atlantic bottlenose dolphin. The white and gray matter are expanded by an abscess (asterisk). H & E. Upper inset: Detail of cerebral obliterative thromboembolism within an abscess. H & E. Lower inset: Filamentous bacteria within a cerebral abscess are highlighted by GMS. GMS (**D**) Liver; Striped dolphin. Multifocal random pyogranulomatous hepatitis with necrosis. Upper inset: Detail of pyogranulomatous inflammation. Lower inset: Filamentous bacteria within intrahepatic inflammatory foci are highlighted by GMS.

Microscopically, most prevalent findings were (by decreasing order): pyogranulomatous and necrotizing bronchopneumonia and pleuritis (5/5; 100%) (Figure 2A,B); pyogranulomatous and necrotizing pulmonary lymphadenitis (5/5; 100%); multiorgan vasculitis and/or vascular fibrinoid necrosis (5/5; 100%); intralesional and intravascular Gram-positive, Ziehl-Neelsen-negative and argyrophilic filamentous bacteria (forming occasional pleomorphic beaded forms and rods) (5/5; 100%); suppurative thromboembolic meningoencephalitis with liquefactive necrosis (3/5; 60%) (Figure 2C); suppurative/pyogranulomatous corticomedullary adrenalitis (3/5; 60%); pyogranulomatous and necrotizing hepatitis (3/5; 60%) (Figure 2D); necrosuppurative splenitis (2/5; 40%); pyogranulomatous and necrotizing nephritis (2/5; 40%); necrosuppurative pharyngeal tonsilitis (2/5; 40%); necrosuppurative pancreatitis (2/5; 40%); pyogranulomatous tracheobronchial/mesenteric lymphadenitis (2/5; 40%); fibrinosuppurative and necrotizing pericarditis (2/5; 40%); necrosuppurative hypophysitis (1/5; 25%); and pyogranulomatous myelitis (1/5; 25%). Severe lymphocytic meningitis with perivascular cuffs (up to 20 cellular layers) and gliosis (1/5; 25%) was seen in case 5 (Table 3).

Additional histologic findings were: lymphoplasmacytic and eosinophilic periportal hepatitis (3/5; 60%); multicentric lymphoid depletion (2/5; 40%); lymphoplasmacytic cholangitis with intracanalicular adult trematodes (2/5; 40%); mesenteric lymph node serosal cavernous hemangioma (1/5; 20%); granulomatous panniculitis, fasciitis, and myositis by *Crassicauda* sp. (1/5; 20%); and chronic lymphoplasmacytic and eosinophilic enteritis with intraluminal cestodes (1/5; 20%).

Bacterial and molecular analyses identified *Nocardia* sp. in 4/4 cases. Because of lack of frozen samples in case 3, nocardiosis was inferred through characteristic histopathologic findings including typical bacterial morphologic features and special tinctorial properties. Isolates from case 1 and 2 were identified by MALDI-TOF MS as *Nocardia farcinica* whereas cases 4 and 5 corresponded to *N. otitidiscaviarum*. Molecular results using 16S rRNA gene sequencing were consistent with the MALDI-TOF identification. Tissues cultured and detailed results are recorded in Table 4. All dolphins but case 3 (frozen samples not available) tested negative for CeMV. Case 5 had concomitant cutaneous herpesvirosis as per histopathologic findings and positive molecular results. 

## 4. Discussion

These results indicate nocardiosis is a significant cause of morbidity and mortality in stranded cetaceans albeit of low occurrence (5/865; [0.58%]) and should be a differential when the typical lesions (pyogranulomas) are present. In agreement with previous observations in humans and animals, the pathologic lesions of nocardiosis in these free-ranging dolphins consisted of multiorgan pleocellular inflammatory lesions that ranged from suppurative to pyogranulomatous with necrosis, intralesional and intravascular filamentous bacteria, vasculitis/fibrinoid wall necrosis and thromboses/thromboemboli. Based on lesion severity and distribution, the disseminated pattern was the most common presentation yet a primary pulmonary form with pulmonary lymph node involvement was detected in all dolphins [12]. This distribution pattern lends support to a major airborne route of entry for *Nocardia* in these cases [12,14,15]. This contrasts with the apparent lack or exceedingly rare evidence of inter-individual transmission of *Nocardia* in infected humans and animals [27]. In this study, other organs such as the brain, liver, adrenal glands, kidney, spleen, and heart were commonly involved. Specifically, two striped dolphins and one bottlenose dolphin presented suppurative thromboembolic meningoencephalitis with liquefactive necrosis by *N. farcinica* (cases 1 and 4) and *N. Otitidiscaviarum* (case 5), respectively. In marine mammals, as well as in other species, cerebral abscesses due to *Nocardia* species are primarily the result of disseminated infection and may occur along with concurrent disease processes [12]. Cerebral abscesses by *N. farcinica* are reported in free-ranging pinnipeds, a captive killer whale and immunocompromised and immunocompetent humans [12,17,28].

Predisposing factors for respiratory nocardiosis are unclear. Other routes of entry, namely inoculation and ingestion have been argued in marine mammals. For instance, primary cutaneous nocardiosis was suspected in a beluga whale and two hooded seals (*Cystophora cristata*) that later developed clinical respiratory signs [12]. Furthermore, presence of *Nocardia* species in fish and shellfish species as well as marine soil makes ingestion another potential route of infection. In such cases, a greater involvement of the gastrointestinal tract as well as liver could be expected; our findings do not support such route of entry in these cases.

Both *N. farcinica* and *N. otitidiscaviarum* have been documented in immunocompromised and immunocompetent individuals causing pulmonary, cutaneous, and systemic infections. Numerous predisposing factors (e.g., human immunodeficiency virus/acquired immunodeficiency syndrome, bone marrow and solid organ transplants, renal insufficiency, hematologic malignancy, immunoglobulin and leukocyte defects) have been associated with compromised cellular immunity and subsequent predisposition for nocardial infection in humans [8]. Canine distemper virus and feline leukemia virus are known predisposing causes in dogs and cats, respectively [29,30]. In this study, poor body condition and lymphoid depletion were morphologic findings that could suggest a debilitated immunosuppressed status in these dolphins. Specifically, a well-known cause of immunosuppression in cetaceans, namely CeMV, was not detected by molecular means in any of the five dolphins. Other potential causes of immunosuppression in cetaceans (e.g., persistent organic pollutants, heavy metals) were not evaluated in this study. Interestingly, all dolphins had mild to moderate cutaneous epibiosis and/or endoparasitism. Parasites are frequently reported in wildlife cetaceans even without pathological significance for the hostage and specific role of parasitism in these cases should be elucidated. Case 5 had cutaneous herpesvirosis, however, no HV genetic material was detected in selected internal organs (data not shown); an immunosuppressive role for HV remains elusive in this case [31]. Further investigations are required to expand knowledge on the influence of immunosuppressive and stressor factors in free-ranging cetaceans and their interplay with bacterioses.

Published accounts of nocardiosis in cetaceans reveal a high prevalence in captive animals, particularly in adults and juveniles, without any evidence of sex predisposition [12,15]. In this study, *N. farcinica* was confirmed in two subadult striped dolphins (cases 1 and 2) and *N. otitiscaviarum* was confirmed in one subadult striped dolphin and a calf bottlenose dolphin (cases 4 and 5). Either in captivity (due to shared enclosure or housing) and in the wild (due to cohesive group behavior), inhalation seems to play a key role for transmission. While inhalation remains a possible route of infection in the calf (case 5), in utero or *ex utero* vertical transmission are also possible yet less likely given the distribution of lesions. Interestingly, *N. farcinica* and *N. otitidiscaviarum* are known causative agents of clinical and subclinical mastitis in dairy cattle usually associated with poor hygienic environmental conditions [30,32]. Furthermore, a captive beluga whale had nocardial mastitis [12,17]. In this study, mastitis was not observed in any of the two female striped dolphins evaluated.

In cetaceans, confirmed *Nocardia* species and host species are *N. asteroides* (in a captive Atlantic bottlenose dolphin, captive killer whale, wild striped dolphin, captive harbor porpoise, and captive pilot whale), *N. farcinica* (in captive killer whale and captive beluga whale), *N. brasilensis* (in a captive Pacific bottlenose dolphin and captive beluga whale), *N. cyriacigeorgica* (in a captive beluga whale), *N. levis* (in a captive Atlantic bottlenose dolphin), *N. otitisdiscaviarum* (in a captive Pacific bottlenose dolphin), and *N. cyriacigeorgica* (in a free-ranging striped dolphin) [12,15,16]. The most common pattern of nocardial infection in cetaceans is the disseminated form with frequent involvement of lungs and lymph nodes [12]. Specifically, in humans and animals, *N. otitidiscaviarum* seems to be less prevalent than other *Nocardia* species owing to its lower prevalence in soil and its presumptive reduced pathogenicity [33,34,35]. Our data suggest *N. otitidiscaviarum* is pathogenic in dolphins.

The pathogenesis of nocardial infections is not fully resolved, particularly the mechanisms of strain-specific presentations. Main pathogenicity factors include tuberculostearic acids, mycolic acids, hydrolytic enzymes, arabinogalactan, catalase and superoxide dismutase enzymes, invasion-like proteins, phospholipase C, hemolysin and the cord factor; secondary metabolites may also play role [36]. Virulent nocardiae inhibit phagosome-lysosome fusion and decrease lysosomal enzyme activity in macrophages, neutralize phagosomal acidification, and even resist the oxidative killing mechanisms of phagocytes [36]. Experimentally, nocardial invasion evokes an early purulent inflammatory response. Subsequently, cell-mediated immunity is triggered by activated macrophages and T-cells cause direct lymphocyte-mediated toxicity to the bacteria. The host ultimately releases antibodies and/or lymphocyte signals, enabling phagocytic cells to kill the organism [36]. Hence, suppurative inflammation prevails in the acute stage, and granulomatous inflammation characterized by predominantly macrophage and T cell infiltration is observed in subacute and chronic stages. In cetaceans, both suppurative-dominated and (pyo)granulomatous-dominated lesions have been reported. In these dolphins, the inflammatory infiltrates ranged from suppurative to pyogranulomatous and lesions ranged from acute to chronic, the latter particularly so in lung and pulmonary lymph nodes.

Reasonable differential diagnoses for nocardiosis in cetaceans include bacterial infections by staphylococci, streptococci, and mycobacteria, as well as mycotic infections by hyphate fungi (mucormycoses, aspergillosis) and systemic yeasts (cryptococcosis, blastomycosis, coccidiodomycosis, histoplasmosis). *Paracoccidioidomycosis ceti* is typically restricted to skin and subcutis. Malignancy, although at lower occurrence in these species, should also be considered grossly. Definitive diagnosis of nocardiosis may rely on cytologic and/or histopathologic findings (intralesional filamentous bacteria with typical tinctorial properties) plus bacterial culture (slow growth) with biochemical properties, and/or molecular analysis (based on 16S rDNA) and sequencing. Serologic methods are of limited value due to common cross-reactivity with other actinomycetes, e.g., *Rhodococcus*, *Streptomyces*, and *Corynebacterium* species as well as hosts immunoreactive for mycobacterial antibodies [37].

The fact that some *Nocardia* species may act as primary pathogens and the growing bacterial antibiotic resistance in aquatic environments, including *Nocardia* species, raises concern for public health. Unfortunately, we did not pursue sensitivity test analyses in our isolates. *Nocardia* species should be considered potential zoonotic agents. Furthermore, timely taxonomic identification allows treatment to begin based on typical susceptibility and pathogenetic traits, which vary between different *Nocardia* species [37]. Clinically relevant *Nocardia* species are classified into 13 antimicrobial susceptibility patterns; *N. farcinica* seems to be a more virulent species, intrinsically resistant to various antibiotics. Rehabilitation centers and dolphinarium should consider prompt culture and sensitivity testing; in numerous occasions, despite long term therapies, late recurrence and eventual death are common sequelae [37]. Coadministration of interferon gamma may enhance response [37].

## 5. Conclusions

In summary, this study ratifies the pathogenic potential of *Nocardia* in free-ranging delphinids. The pathologic signature of nocardiosis in these dolphins consisted of multiorgan pleocellular inflammatory lesions that ranged from suppurative to pyogranulomatous with necrosis, intralesional and intravascular filamentous bacteria and vasculitis/fibrinoid wall necrosis. To the authors’ knowledge, these represent the first accounts of *N. farcinica* and *N. otitidiscaviarum* in free-ranging cetacean species, specifically from the central eastern Atlantic Ocean and the western Mediterranean Sea. Nocardial infections should be considered in the differential diagnosis of pyogenic to pyogranulomatous lesions in free-ranging cetaceans. Caution is advised when performing postmortem examinations in such cases due to potential for zoonotic transmission.

## Figures and Tables

**Table 1:** Biologic and stranding epidemiology data for delphinids included in this study.

Case	Case No.	Species	Stranding Location	Gender	Age	BC	DC	ND
297_14	1	SD	Almeria	F	adult	3	2	19 March 2014
292_14	2	SD	Cádiz	F	adult	3	2	22 April 2014
34_16	3	SD	Málaga	M	adult	1	2	11 November 2015
148_01	4	SD	Gran Canaria	M	adult	4	2	26 November 2001
1103_20	5	ABD	Gran Canaria	M	Calf	1	2	14 June 2020

SD = striped dolphin; ABD = Atlantic bottlenose dolphin; BC = body condition (1: emaciated, 2: poor, 3: moderate, 4: good); DC = decomposition status (1: very fresh, 2: fresh, 3: moderate autolysis; 4: advanced autolysis); ND = necropsy date.

**Table 2:** Most prevalent gross findings in dolphins included in this study.

Gross Findings	Af/Ev (%)
Pulmonary pyogranulomatous lymphadenitis (lymphadenomegaly)	5/5 (100)
Pleural, pulmonary, and bronchial pyogranulomas	5/5 (100)
Cerebral abscess	3/5 (60)
Disseminated nodules across liver capsule and/or parenchyma	3/5 (60)
Adrenal gland pyogranulomas	3/5 (60)
Pyogranulomatous pericarditis	2/5 (40)
Peritoneal (serosal) pyogranulomas	3/5 (60)
Endotracheal mucosal pyogranulomas	2/5 (40)
Pharyngeal (tonsil) pyogranulomas	2/5 (40)
Splenic pyogranulomas	2/5 (40)
Mesenteric, tracheobronchial pyogranulomatous lymphadenitis (lymphadenomegaly)	2/5 (40)
Renal pyogranulomas	2/5 (40)
Pancreatic pyogranulomas	1/5 (25)
Hypophyseal pyogranulomas	1/5 (25)

Af = affected animals; Ev = evaluated animals.

**Table 3:** Most prevalent microscopic findings in dolphins included in this study.

Microscopic Findings	Af/Ev (%)
Pyogranulomatous and necrotizing bronchopneumonia and pleuritis	5/5 (100)
Pyogranulomatous and necrotizing pulmonary lymphadenitis	5/5 (100)
Multiorgan vascular vasculitis/fibrinoid necrosis	5/5 (100)
Intralesional filamentous bacteria	5/5 (100)
Suppurative thromboembolic meningoencephalitis with necrosis	3/5 (60)
Suppurative/pyogranulomatous cortical/medullar adrenalitis	3/5 (60)
Pyogranulomatous and necrotizing hepatitis	3/5 (60)
Necrosuppurative splenitis	2/5 (40)
Pyogranulomatous and necrotizing nephritis	2/5 (40)
Necrosuppurative pharyngeal tonsilitis	2/5 (40)
Necrosuppurative pancreatitis	2/5 (40)
Pyogranulomatous tracheobronchial/mesenteric lymphadenitis	2/5 (40)
Fibrinosuppurative and necrotizing pericarditis	2/5 (40)
Necrosuppurative hypophysitis	1/5 (25)
Lymphocytic meningitis with perivascular cuffs and gliosis	1/5 (25)
Pyogranulomatous mielitis	1/5 (25)
Pyogranulomatous (aortic) arteritis	1/5 (25)

Af = affected animals; Ev = evaluated animals.

**Table 4:** Microbiologic findings in dolphins included in this study.

Case No.	Lung	Pleura	Liver	Kidney	Spleen	Peritoneum (Serosae)	Heart	Pulmonary (LN)	Adrenal gland	Pharyngeal Tonsil	Pancreas	Trachea	Hypophysis	Brain	Other Lymph Nodes	Organism
1	+++	++	++	?	+++	++	++	+++	+++	+	-	+++	+++	+	+ (PS); + (MS)	*N. farcinica*
2	+++	-	-	-	-	+	-	+++	-	++	-	++	-	-	-	*N. farcinica*
3	+++	-	++	++	-	-	-	++	++	-	-	-	-	-	-	Nocardia sp.*
4	+++	++	+	+++	++	+	++a	+++	++	-	+++	-	-	+++	-	*N. otitidiscaviarum*
5	+++	-	-	-	-	-	-	+++	-	-	-	-	-	+++	+++ (TBr)	*N. otitidiscaviarum*

^a^ Pericardium. * Presumed histological and histochemical diagnosis. + = mild; ++ = moderate; +++ = severe.

## Data Availability

The data presented in this study are available on request from the corresponding author.
